# Framing Bias in the Interpretation of Quality Improvement Data: Evidence From an Experiment

**DOI:** 10.15171/ijhpm.2019.08

**Published:** 2019-03-02

**Authors:** Andrew Ballard

**Affiliations:** School of Public Affairs and Administration, Rutgers University, Newark, NJ, USA.

**Keywords:** Quality Improvement, Performance Management, United States, Framing, Data Interpretation

## Abstract

**Background:** A growing body of public management literature sheds light on potential shortcomings to quality improvement (QI) and performance management efforts. These challenges stem from heuristics individuals use when interpreting data. Evidence from studies of citizens suggests that individuals’ evaluation of data is influenced by the linguistic framing or context of that information and may bias the way they use such information for decision-making. This study extends prospect theory into the field of public health QI by utilizing an experimental design to test for equivalency framing effects on how public health professionals interpret common QI indicators.

**Methods:** An experimental design utilizing randomly assigned survey vignettes is used to test for the influence of framing effects in the interpretation of QI data. The web-based survey assigned a national sample of 286 city and county health officers to a "positive frame" group or a "negative frame" group and measured perceptions of organizational performance. The majority of respondents self-report as organizational leadership.

**Results:** Public health managers are indeed susceptible to these framing effects and to a similar degree as citizens. Specifically, they tend to interpret QI information presented in a "positive frame" as indicating a higher level of performance as the same underlying data presenting in a "negative frame." These results are statistically significant and pass robustness checks when regressed against control variables and alternative sources of information.

**Conclusion:** This study helps identify potential areas of reform within the reporting aspects of QI systems. Specifically, there is a need to fully contextualize data when presenting even to subject matter experts to reduce the existence of bias when making decisions and introduce training in data presentation and basic numeracy prior to fully engaging in QI initiatives.

## Background

Public agencies are very good at collecting data, and this is particularly true in health departments. Initiatives like the 10 essential services and accreditation have dramatically expanded the use of data to improve decision-making.^1^ Quality improvement (QI) refers to the practice of continuously evaluating programmatic operations and managerial practices to improve all aspects of service delivery and effectiveness.^2,3^ The term QI is often used interchangeably with the term ‘performance management’ as the foundations of QI and performance management are largely similar.^2,4,5^ A core feature of QI is the collection of timely and reliable data, however, research has revealed a gap between adopting (collecting data) and actual use (integrating data into decision-making process).^6,7^

One potential explanation for this lack of real information use is the way in which individuals see and interpret such information. In the broad field of public management, a growing body of literature has explored how citizens and practitioners evaluate quantitative information to test for biases and potential areas of reporting improvement.^8-11^ Specifically, one challenge to QI is the way individuals view statistically equivalent but contextually different information, often referred to as equivalency framing or prospect theory.^12^

This study seeks to extend existing literature on the field of prospect theory into public health QI by examining how public health professionals view and interpret common QI data. A brief review of relevant literature is provided followed by an experimental study involving the evaluation of various statistical equivalency scenarios by public health professionals.

### Performance Information in Public Health

Existing QI literature often focuses on the internal dynamics of health departments and how they contribute to performance.^2,3,13-15^ However, fewer studies examine information use as a cognitive process for the purposes of performance and QI. It is clear from other fields of study that simply having operational data does not ensure performance improvement. An important potential contributor to this is the limitation various important stakeholder groups demonstrate when interpreting information.

For some public managers, it appears that social norms within the organization and pre-existing cognitive states have a greater influence on whether performance information is used than the technical “quality” of the data.^16^ The framing and direction of performance information have been shown to influence both how elected politicians interpret results and their attitudes towards spending and reform.^17,18^ Citizens attitudes have also been shown to be sensitive to variations in the way government data is reported, particularly in the area of framing effects and motivated reasoning.^19,20^ Finally, research on performance information acceptance by front-line employees (teachers) suggests that such data is more likely to be accepted if it shows the organization is performing well.^21^

These studies are important to the way we think about performance management and QI in public health. Good governance and advocacy groups like the Public Health Accreditation Board promote the collection and use of operational data to assist in decision-making.^22,23^ However, one challenge faced by public health agencies is the lack of a uniform QI model within the field.^14^ In other fields, such as transportation services, sanitation, and others, the “performancestat” model has emerged as a primary vehicle by which agencies track and discuss organizational data.^24^ There is a need for a strong conceptual framework for what performance measurement and QI means to public health organizations and how agencies can link their activities to that notion of performance.^25^ Vital to this, is a thorough consideration of how information is presented so that accurate interpretations can be made.

### Framing Effects in Information Cognition

Within psychology, prospect theory suggests that the point of reference individuals use to judge whether the information is perceived as a benefit or harm to some individual or organization can be shifted by framing the information in a positive or negative light.^26^ That is, if you present probabilistically equivalent information in a positive light it will be interpreted differently than it would had it been presented it in a negative light.^12^ This notion of equivalency framing has been expanded and adopted in experimental research to explore numerous fields ranging from public management to health behavior.

The very concept of QI relies on an assumption that information is objective and decision-makers are able to receive operational reports and design policy reforms around those. The flaw in this assumption is that we know, through decades of psychological research, that information has a fluid meaning depending on the context and presentation. By changing the valence of a particular data point, you can change the meaning individuals derive from it.^27^ For example, consumers have been shown to rate a product higher if presented with product information in a positive frame in contrast to consumers presented the same product and information in a negative frame.^28^

Other fields have shown a relationship between informational valence and comprehension. For example, when patients are presented with behavioral risk factors as relative ratings among a series of potential behaviors, they tend to perceive that information as more useful than information presented as absolute risk.^29^ A previous study utilizes Danish citizens to measure the perceptual shift of performance information. It shows that citizens’ initial evaluation of public performance information is highly susceptible to framing effects.^20^ Similarly, citizen evaluation of performance information is also influenced by the physical positioning of the information, specifically a left-most digit bias in information preferences.^30^ Equivalency framing in the field of public health management, however, is relatively underexplored compared to other fields.

**H1–** Placing public health performance target data in a positive frame will result in a higher rating of organizational performance than performance data placed in a negative frame.

**H2–** Placing public health training and outreach data in a positive frame will result in a higher rating of organizational performance than training and outreach data placed in a negative frame.

## Methods

### Sampling

This study uses a national sample of US local and county public health managers (Health Officers). There exists a long history of data collection and analysis within public health yet this sub-set of the public service has been rather underexplored in terms of information display and use relative to generalist public managers.^13^ The survey frame is a national list of health departments maintained by the National Association for County and City Health Officers (NACCHO). This frame and subsequent sample are appropriate for this particular study because the NACCHO list of health departments is the most comprehensive catalog of all local and county health department contact information readily available for use. Importantly, this list is not simply an association membership list, but a comprehensive list of each local and county health department and contact information for organizational leadership. This prevents any biases which may result from an opt-in membership list.

A mixed-mode approach to contacting individual participants was employed in this study. Specifically, a strategy often referred to as mail push-to-web.^31^ In this strategy, individuals were mailed an introduction letter that described the purpose of the study and why their participation is important. Three days later, they were sent an electronic request for participation via email that included a clickable link directing them to the questionnaire. The email contact strategy is a modified variant of the recommended timeline presented by Dillman et al.^32^ As expected, very few individuals responded through the direct letter, with the vast majority responding via the email requests. A total of 344 individuals responded to the survey in some form and after removing incomplete responses, a final response rate of 286 individuals or 11% is observed.

### Experimental Design

The use of experimental survey vignettes is a popular approach to examining causal relationships between a treatment of some sort and individual perceptions.^33^ Several studies have indeed validated the approach as a relatively robust tool that can illustrate real-world behavior given sufficient design rigor.^34^ Typically, the process involves presenting each experimental group with the same initial information and making a small number of changes throughout to isolate the treatment effect from other design effects. The fewer the changes in the presentation between each group, the stronger the causal inference can be.^35^

In this study, individuals are placed into 2 general experimental groups, a “positive framing” group, and a “negative framing” group. They are then exposed to 2 separate presentations of performance information from a hypothetical local public health department. The types of QI information presented are: (1) a summary statement of performance target achievement, and (2) citizen satisfaction with an emergency preparedness training and outreach event held by a local public health department.

**Figure 1:**
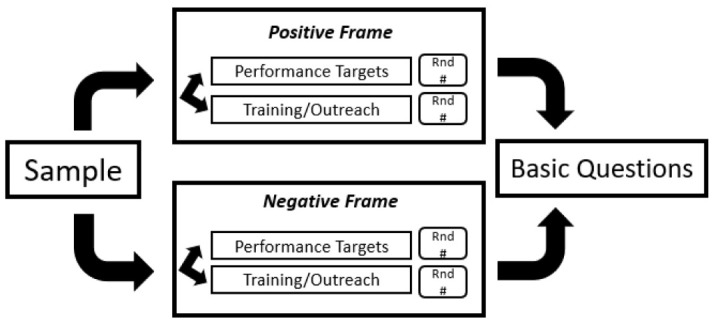
Framing Effects Experimental Design.

The treatment occurs when participants are exposed to different information “frames,” meaning when the information is presented in a positive light or a negative light. For example, some participants may see “….successfully achieved 90% of their targets” while others may see “….did not meet 10% of their performance targets.” The underlying reality of organizational performance is the same; however, the context of the information changes thus influences individual perceptions. Figure 1 illustrates the overall experimental design. After the performance information is presented, participants are asked to evaluate the hypothetical agency’s performance on a linear scale of 0 to 100, with 0 being the worst possible performance and 100 being the best possible performance.

As shown in Figure 1, there are three points of randomization participants experience when completing the questionnaire, all performed by the survey software (Qualtrics). The first is when they are assigned to the “positive” group or the “negative” group. Once in these groups, they view both vignettes of the same frame and respond to each. This randomization is meant to achieve statistical equivalency between the 2 treatment groups. The second point of randomization is the order in which they see the vignettes. Some randomly see the performance targets presentation first while others will see the training and outreach presentation first. This is meant to control away from any unintended question ordering effects.^36^ Finally, within each vignette, participants are randomly assigned a number used to describe the key performance indicator. For example, positive framing respondents see a number attached to their vignette between 80%-95% (*X*_p_ = 80, 95), while negative framing respondents see a random number between 5%-20% (*X*_n_ = 5, 20). The purpose of this randomization is twofold: first to attempt to reduce effects of common source bias that are inherent in survey research,^37^ and second to test whether the severity or level of the frame influences the way individuals perceive the framed information. Table 1 presents the full text of the experimental vignettes.

**Table 1:** Treatment Frames and Vignette Wording

**Treatment Frame**	**Treatment Wording**
Positive	The 2016 profile report from the NACCHO suggests that the majority of health departments develop measurable performance and QI objectives. Suppose one of these departments successfully met (*X*_p_ = 80, 95) percent of its performance objectives. How would you rate the performance of this department? (Scale 0-100) (n = 147)
	The 2016 profile report from the NACCHO suggests that the majority of health departments provide emergency preparedness training to members of their community. Suppose one of these departments conducted a post-training survey and (*X*_p_ = 80, 95) percent of attendees were satisfied with the event. How would you rate the performance of this department? (Scale 0-100) (n = 147)
Negative	The 2016 profile report from the NACCHO suggests that the majority of health departments develop measurable performance and QI objectives. Suppose one of these departments did not meet (*X*_n_ = 5, 20) percent of its performance objectives. How would you rate the performance of this department? (Scale 0-100) (n = 139)
	The 2016 profile report from the NACCHO suggests that the majority of health departments provide emergency preparedness training to members of their community. Suppose one of these departments conducted a post-training survey and (*X*_n_ = 5, 20) percent of attendees were unsatisfied with the event. How would you rate the performance of this department? (Scale 0-100) (n = 139)

Abbreviations: NACCHO, National Association of County and City Health Officers; QI, quality improvement.

The randomization process is meant to produce statistically equivalent treatment groups, meaning groups that have similar makeup in terms of important individual characteristics. Statistical equivalency helps control for any variation in responses due to influences other than the experimental treatment, such as gender or position.^38^ Balance tests comparing the 2 experimental groups suggest there is no systematic difference between those receiving graphical information and those receiving numerical information^ [1] ^. This includes common demographic information as well as reported levels of familiarity with both using performance information. The level of experience with performance management and the level of experience with training and outreach activities were measures to obtained as a possible “alternative source of information” that may inform these individual evaluations beyond simply the framing treatment.^20^ A high level of experience with performance management may act as a moderator for the positive and negative treatments, just as a high level of experience with training and outreach may moderate the effects of the framing treatment.

## Results

Table 2 provides a series of descriptive statistics for the survey sample. In addition, I compare this study’s sample to a much larger profile survey administered by the NACCHO. The 2016 “NACCHO Profile” survey achieved a 76% response rate or 1930 respondents. This is meant to provide rough evidence for generalizability. The sample is a majority female, majority white, and rather highly education with nearly two-thirds possessing and master’s degree or higher. Agencies serving fewer than 50 000 residents made the largest subset of the sample. There are few large variations between this study’s sample and the larger national except in the population served. This study’s sample is slightly more skewed towards larger populations than the NACCHO findings.

**Table 2:** Survey Sample Descriptive Statistics

	**n**	**N**
Gender		
Female	58%	62%
Male	42%	38%
Age		
<40	15%	12%
40-49	19%	24%
50-59	36%	39%
60-69	27%	24%
70 or older	3%	2%
Education level		
Associates	6%	8%
Bachelors	30%	30%
Masters	49%	46%
PhD/JD	15%	15%
Experience		
< 1 year	1%	
1-3 years	8%	
4-5 years	12%	
6-10 years	8%	
11-20 years	28%	
20 years+	42%	
Population		
<50 000	48%	61%
50 000-499 999	38%	33%
500 000+	14%	6%
Race		
White	88%	90%
Non-White	9%	8%
Hispanic/Latino	3%	2%

n = 286, N = 1930, 2016 NACCHO Profile Survey.

The vast majority of respondents, 84%, identified primarily as the administrative head of the department, often referred to as the “health officer.” However, some research suggests that performance-related responsibilities are dispersed throughout the organization, and thus having a mix of positions is important for exposing the nuances in perceptions.^39^ For many smaller health departments, the health officer fills numerous roles in the organization. 15% of the sample reported performing other duties in addition to their leadership responsibilities, these duties include education, environmental health, inspections, and epidemiology.

There is a limited ability to determine the random or systematic nature of the nonresponse rate in this study. This is due to the unavailability of individual-level data for the entire sample frame, preventing a *t* test. However, comparing aggregate figures shows very few instances where a rather large difference exists between the sample and the sample frame across important categories.

**Figure 2:**
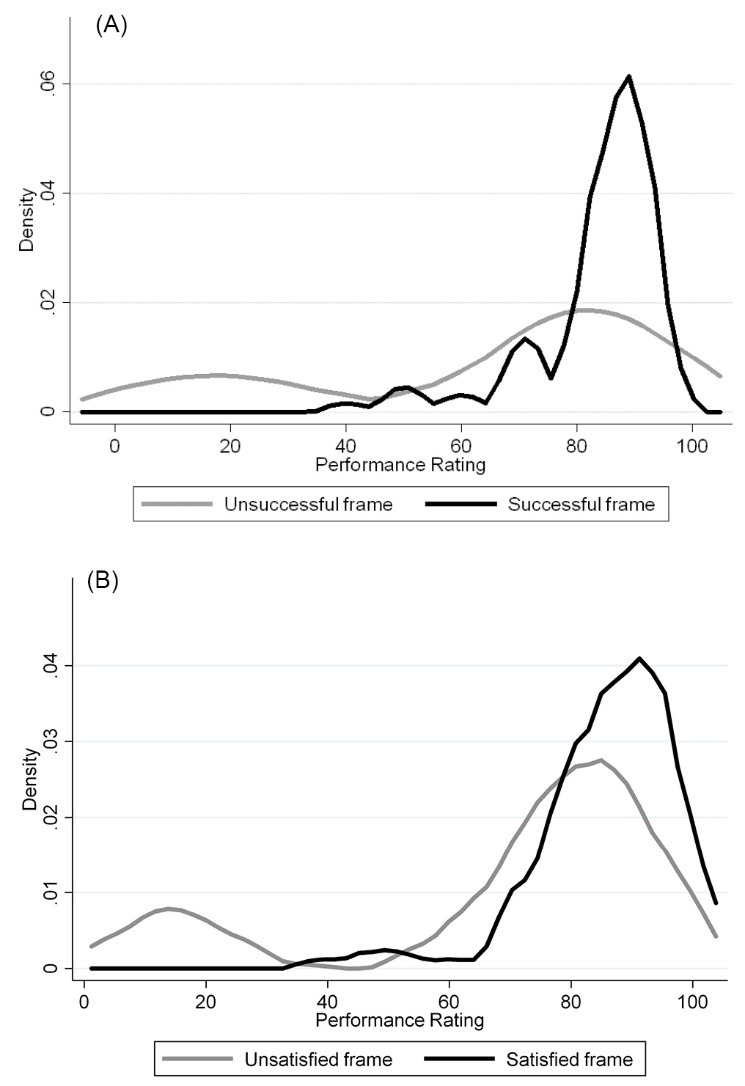
Density Plot of Performance Target Treatment (A) Training (B).

The first series of results of the experimental treatment are provided in Figures 2A and 2B. Density plots are shown, representing the frequency of responses across the total 0-100 distributions for the positive (successful/satisfied) and negative (unsuccessful/unsatisfied) frame groups. Each category — performance targets and training — are combined into one density plot to compare the responses for the positive (successful/satisfied) and negative (unsuccessful/unsatisfied) frames.

Both figures suggest a greater density of responses at the higher performing end of the distribution by those individuals presented with a positive framed treatment. In Figures 2A, which illustrates the responses to the performance targets vignette, those in the positive frame exhibit a pronounced spike in density at the high end of the distribution, while those in the negative framing group show a rather subtler spike at the high end with a larger density towards the poor performance end of the distribution. Figure 2B shows the responses to the training and outreach vignette, and there is a clear difference in responses between the positive and negative framing groups, with the positive framing respondents reporting higher performance while there is a spike in density towards the low end of the distribution by those in the negative framing group.

Robustness tests are proved in Tables 3 and 4. Ordinary least squares regression is used to test the effects of the main treatment while controlling for alternative influences on data interpretation. The continuous variable (0-100) response individuals gave to the post-vignette question is the primary outcome variable included in the model. The primary independent variable is a binary indicator of which treatment group to which respondents were assigned with 1 representing those in the positive frame group (successful/satisfied) and 0 representing those in the negative frame group (unsuccessful/unsatisfied). The “treatment percent” variable is constructed by taking the difference between the individual random numbers assigned and the mean random number assigned to the entire sample.

**Table 3:** Ordinary Least Squares Regress Results for Performance Target Vignette

** **	**Model A**	**Model B**	**Model C**	**Model D**	**Model E**
Framing (1 = successful)	19.58***	19.74***	19.72***	19.72***	19.73***
	(3.43)	(3.42)	(3.38)	(3.39)	(3.41)
Treatment percent		-0.53*	-1.34*	-1.35*	-1.35*
		(0.42)	(0.56)	(0.57)	(0.57)
Frame*treatment percent			1.76*	1.80*	1.80*
			(0.84)	(0.84)	(0.85)
Performance experience				-0.47	-0.47
				(1.69)	(1.69)
Education					0.12
					(2.19)
Intercept	63.94***	63.86***	63.74***	65.31***	64.76
Adjusted R^2^	0.16	0.16	0.18	0.17	0.16
F-statistic	32.70	17.16	13.21	9.87	7.85
N	286	286	286	277	275

Note: Standard error in parenthesis. *** denotes *P* < .001, * denotes *P* < .05. Model E also includes control variables for gender, in a leadership position, population served, and the number of employees.

**Table 4:** Ordinary Least Squares Regress Regress Results for Training Vignette

	**Model A**	**Model B**	**Model C**	**Model D**	**Model E**
Framing (1 = Satisfied)	15.99***	16.09***	16.08***	16.08***	16.02***
	(3.15)	(3.16)	(3.15)	(3.17)	(3.18)
Treatment percent		-0.32	-0.64	-0.66	-0.65
		(0.39)	(0.53)	(0.53)	(0.53)
Frame*treatment percent			0.69	0.71	0.72
			(0.78)	(0.79)	(0.79)
Training experience				-0.65	-0.63
				(1.57)	(1.53)
Education					-0.69
					(2.05)
Intercept	69.79***	69.73***	69.70***	71.89***	74.99***
Adjusted R^2^	0.13	0.12	0.12	0.12	0.11
F-statistic	25.74	13.19	9.04	6.79	5.43
N	286	286	286	277	275

Notes: Standard error in parenthesis. *** denotes *P* < .001. Model E also includes control variables for gender, in a leadership position, population served, and the number of employees.

Across all the models and both experiments, the main treatment effect is statistically significant at the *P* < .001 level. This suggests that a positive framing of information results in public health professionals assigning a higher evaluation of performance to that organization. Additionally, these studies show no relationship between alternative sources of information and the overall treatment effect.

**Figure 3:**
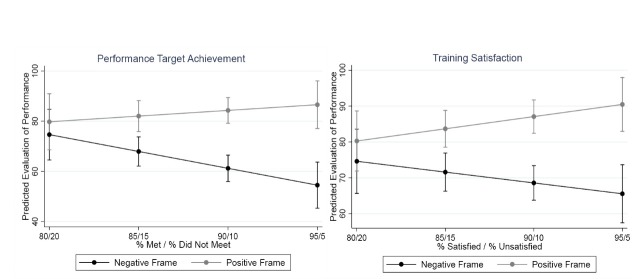
Ordinary Least Squares Predictions for Performance Targets (Left) and Training Vignettes (Right).

Figure 3 provides ordinary least squares estimated slopes for positive and negative frames, specifically, they should the effect of changing the percentage shown to respondents. The light grey line represents the predicted level of performance individuals will report for positive framed indicators from 80% to 95%. The black line represents the predicted level of performance individuals will report for negatively framed indicators from 5% to 20%. For example, when viewing a training vignette, an individual presented with a reported satisfaction rate of 95% will report a roughly 90% level of organizational performance as indicated by the grey line. In the same graph, now viewing the black line, an individual shown a 5% level of dissatisfaction will report a roughly 66% level of organizational performance.

## Discussion and Conclusions

In this sample of local public health professionals, positively framed operational data will result in a more positive assessment of organizational performance, and the opposite for negatively framed data. These findings have important implications for the reporting of performance and QI information in public health. Much of the existing research in performance highlights the importance of reporting in a QI framework^40^; this paper, however, sheds lights on potential shortcomings public health agencies should avoid.

An important finding in the lack of a major deviation from the treatment effect size observed in previous studies using citizens as the sample. In a 2015 paper by Asmus Olsen using Danish citizens, the effect size expressed by non-subject-matter experts is very similar to that found in this sample of health professionals.^20^ This provides a rather interesting source of deliberation. Presumably, those working in public health agencies have received more specialized training in QI, performance information, and general numeracy, and therefore would be expected to exhibit less vulnerability in terms of framing effects. However, this study does not support this hypothesis; public health professionals experience a similar level of sensitivity to framing effects as do citizen samples even while controlling for those who report higher experience in performance and training activities. Additionally, the Olsen findings suggest that “alternative sources of information” like familiarity with the related subject-matter moderated the effect of negative framed information. This paper does not confirm this finding. Perhaps this is simply an artifact in this paper’s sample or caused by some other phenomenon.

This might suggest a possible weakness in the current state of performance in public health and that to truly adopt such a system, additional specialized training in the area is needed. It suggests that collecting and reporting operational information to decision-makers does not necessarily mean that it will be interpreted accurately, or that the most appropriate decision will be made. Advocacy of performance management and QI systems may wish to expand their guidance to include things like data visualization and reporting training, or techniques for discussing and deliberating the findings of program analysis.

These results suggest a need to fully contextualize information when it is being presented to even a professional audience so as to avoid less than optimal decision-making. Perhaps a solution to this issue is to provide both a positive and a negative frame when presenting such data. For example, rather than saying “this unit has successfully achieved 80% of its targets” or “this unit failed to meet 20% of its targets,” combining these into one singular statement of fact may provide important nuance and robustness to the information. Evidence from business management and general data cognition suggest that data visualization may provide a possible remedy for this bias. The effective use of visual aids has been shown to simplify and clarify complex numerical information.^41,42^

Overall, this type of research is valuable to the study and indeed practice of QI. It begs the question, what other biases are public health professionals susceptible to when interpreting important operational data? Other fields have explored issues of numeracy, motivated reasoning, and choice complexity but research of this type in the field of public health administration is sparse.^30,43-45^ To better understand how QI systems can improve how public health agencies make decisions, it may be important to explore these issues in the health context.

It may also provide insights into one possible cause of the symbolic adoption observed in previous performance-related literature, whereby organizations collect operational data but show little evidence of integrating it into their decision-making process.^7^ Many scholars have suggested the difficulty in actually using QI information to make decisions beyond simply collecting routine organizational information.^6,7,39,46^ The routine of reporting performance information is a critical component to this process and identifying best practices in this regard may help eliminate the adoption-utilization gap.

Several important limitations should bound the conclusions drawn from these results. “Successfully met” and “did not meet” may not be precisely logical opposites. It’s unclear whether this moderates or exaggerates the results. The inclusion of “failed to meet” may further diverge the responses for the vignettes. There is also a limitation to the experimental method employed in this study. As the situation presented to respondents is hypothetical, the risk and incentives associated with completing this task are relatively low compared to a real-world situation. Individuals may spend more time deliberating a similar situation if presented in their actual organization. This may reduce the real-world effects of framing effects. Additionally, no true control group is used in this experiment. A control group may have consisted of individuals being presented both the positive and negative frames for each vignette. This omission of this type of control group was done both to align with previous similar studies as well as ensuring a sufficient sample size for the experiment.

## Ethical issues

The Rutgers University Institutional Review Board reviewed the study protocol and classified it as exempt from full board review.

## Competing interests

Author declares that he has no competing interests.

## Author’s contribution

AB is the single author of the paper.

## Endnotes


[1] To test for any imbalances between the two experimental groups, a logit regression model was constructed using a dichotomous dependent variable for treatment and control and the scaled demographic questions as independent variables.


## Key messages

**Implications for policy makers**

- The existence of quality improvement (QI) data, even when complete and timely, does not guarantee the effective use of that information to make decisions. Awareness of cognitive limitations and biases in decision-makers is important.
- The same underlying information can be seen very differently by decision-makers depending on how you frame it. Provide a full context for reported performance targets to reduce decision-makers’ likelihood of misinterpreting information.

**Implications for public**

Public health professionals show a similar level of susceptibility to cognitive bias when interpreting organizational data as citizens. Although the use of quality improvement (QI) data in public health agencies has expanded in recent decades, this research suggests that a greater emphasis on appropriate context in these reports is needed. Additionally, this shows that public health professionals attempting to adopt and implement a data-oriented management system need to expand their quantitative skills and analysis competencies to fully utilize evidence to make decisions and design policy.

## References

[1] Corso LC, Wiesner PJ, Halverson PK, Brown CK (2000). Using the essential services as a foundation for performance measurement and assessment of local public health systems. J Public Health Manag Pract.

[2] Riley WJ, Moran JW, Corso LC, Beitsch LM, Bialek R, Cofsky A (2010). Defining quality improvement in public health. J Public Health Manag Pract.

[3] McLees AW, Nawaz S, Thomas C, Young A (2015). Defining and assessing quality improvement outcomes: a framework for public health. Am J Public Health.

[4] Behn RD (2003). Why measure performance? Different purposes require different measures. Public Adm Rev.

[5] Jakobsen ML, Baekgaard M, Moynihan DP, van Loon N (2017). Making sense of performance regimes: Rebalancing external accountability and internal learning. Perspectives on Public Management and Governance.

[6] de Lancer Julnes P, Holzer M (2001). Promoting the utilization of performance measures in public organizations: An empirical study of factors affecting adoption and implementation. Public Adm Rev.

[7] Moynihan DP. The dynamics of performance management: Constructing information and reform. Georgetown University Press; 2008.

[8] James O, Jilke SR, Van Ryzin GG. Experiments in public management research: Challenges and contributions. Cambridge University Press; 2017.

[9] Baekgaard M, Christensen J, Dahlmann CM, Mathiasen A, Petersen NBG (2017). The role of evidence in politics: Motivated reasoning and persuasion among politicians. Br J Polit Sci.

[10] Hjortskov M, Andersen SC (2015). Cognitive biases in performance evaluations. J Public Adm Res Theory.

[11] Marvel JD (2015). Unconscious bias in citizens’ evaluations of public sector performance. J Public Adm Res Theory.

[12] Kahneman D, Tversky A (1979). Prospect theory: An analysis of decision under risk. Econometrica.

[13] Erwin PC (2008). The performance of local health departments: a review of the literature. J Public Health Manag Pract.

[14] Handler A, Issel M, Turnock B (2001). A conceptual framework to measure performance of the public health system. Am J Public Health.

[15] Mauer BJ, Mason M, Brown B (2004). Application of quality measurement and performance standards to public health systems: Washington State’s approach. J Public Health Manag Pract.

[16] Kroll A (2015). Explaining the use of performance information by public managers: A planned-behavior approach. Am Rev Public Adm.

[17] Baekgaard M, Nielsen PA (2013). Performance information, blame avoidance, and politicians’ attitudes to spending and reform: Evidence from an experiment. J Public Adm Res Theory.

[18] George B, Baekgaard M, Decramer A, Audenaert M, Goeminne S (2018). Institutional isomorphism, negativity bias and performance information use by politicians: A survey experiment. Public Adm.

[19] Baekgaard M, Serritzlew S (2016). Interpreting performance information: Motivated reasoning or unbiased comprehension. Public Adm Rev.

[20] Olsen AL (2015). Citizen (dis) satisfaction: An experimental equivalence framing study. Public Adm Rev.

[21] Jakobsen M, Petersen NBG, Laumann TV (2018). Acceptance or Disapproval: Performance Information in the Eyes of Public Frontline Employees. J Public Adm Res Theory.

[22] Accreditation Background. PHABOARD.org. https://www.phaboard.org/accreditation-background/. Accessed February 1, 2017.

[23] Carman AL, Timsina L (2015). Public health accreditation: rubber stamp or roadmap for improvement. Am J Public Health.

[24] Behn RD. The PerformanceStat potential: A leadership strategy for producing results. Brookings Institution Press and Ash Center for Democratic Governance and Innovation; 2014.

[25] Donabedian A. The definition of quality and approaches to its assessment. Exploration in quality assessment and monitoring. Ann Arbor: Health Administration Press; 1980.

[26] Kahneman D, Tversky A (2013). Prospect theory: An analysis of decision under risk Handbook of the fundamentals of financial decision making: Part I. World Scientific.

[27] Levin IP, Schneider SL, Gaeth GJ (1998). All frames are not created equal: a typology and critical analysis of framing effects. Organ Behav Hum Decis Process.

[28] Levin IP, Gaeth GJ (1988). How consumers are affected by the framing of attribute information before and after consuming the product. J Consum Res.

[29] Edwards A, Elwyn G, Covey J, Matthews E, Pill R (2001). Presenting risk information--a review of the effects of “framing” and other manipulations on patient outcomes. J Health Commun.

[30] Olsen A (2013). Leftmost-digit-bias in an enumerated public sector? An experiment on citizens’ judgment of performance information. Judgm Decis Mak.

[31] Weiner MD, Puniello OT, Noland RB (2016). Conducting Efficient Transit Surveys of Households Surrounding Transit-Oriented Developments. Transp Res Rec.

[32] Dillman DA, Smyth JD, Christian LM. Internet, phone, mail, and mixed mode surveys: The tailored design method. John Wiley & Sons; 2014.

[33] Atzmuller C, Steiner PM (2010). Experimental vignette studies in survey research. Methodology.

[34] Hainmueller J, Hangartner D, Yamamoto T (2015). Validating vignette and conjoint survey experiments against real-world behavior. Proc Natl Acad Sci U S A.

[35] Alexander CS, Becker HJ (1978). The use of vignettes in survey research. Public Opin Q.

[36] Groves RM, Fowler FJ Jr, Couper MP, Lepkowski JM, Singer E, Tourangeau R. Survey Methodology. John Wiley & Sons; 2011.

[37] Meier KJ, O’Toole LJ, Jr Jr (2012). Subjective organizational performance and measurement error: Common source bias and spurious relationships. J Public Adm Res Theory.

[38] Remler DK, Van Ryzin GG. Research methods in practice: Strategies for description and causation. Sage Publications; 2010.

[39] Holzer M, Ballard A, Kim M, Peng S, Deat F (2019). Obstacles and opportunities for sustaining performance management systems. Int J Public Adm.

[40] Moynihan DP, Lavertu S (2012). Does involvement in performance management routines encourage performance information use? Evaluating GPRA and PART. Public Adm Rev.

[41] Elting LS, Martin CG, Cantor SB, Rubenstein EB (1999). Influence of data display formats on physician investigators’ decisions to stop clinical trials: prospective trial with repeated measures. BMJ.

[42] Ogiela L, Ogiela MR. Cognitive techniques in visual data interpretation. Springer; 2009.

[43] Kahan DM (2013). Ideology, motivated reasoning, and cognitive reflection: An experimental study. Judgm Decis Mak.

[44] Hvidman U, Andersen SC (2016). Perceptions of public and private performance: Evidence from a survey experiment. Public Adm Rev.

[45] Marvel JD (2015). Public opinion and public sector performance: Are individuals’ beliefs about performance evidence-based or the product of anti–public sector bias?. International Public Management Journal.

[46] Cavalluzzo KS, Ittner CD (2004). Implementing performance measurement innovations: evidence from government. ‎Account Organ Soc.

